# Finish line distinctness and accuracy in 7 intraoral scanners versus conventional impression: an in vitro descriptive comparison

**DOI:** 10.1186/s12903-018-0489-3

**Published:** 2018-02-23

**Authors:** Robert Nedelcu, Pontus Olsson, Ingela Nyström, Andreas Thor

**Affiliations:** 1 Department of Surgical Sciences, Plastic & Oral and Maxillofacial Surgery, Uppsala University, 751 85 Uppsala, Sweden; 2 Department of Information Technology, Centre for Image Analysis, Uppsala University, Uppsala, Sweden

**Keywords:** Accuracy, Digital impression, Finish line, Intraoral scanner, 3D compare analysis

## Abstract

**Background:**

Several studies have evaluated accuracy of intraoral scanners (IOS), but data is lacking regarding variations between IOS systems in the depiction of the critical finish line and the finish line accuracy. The aim of this study was to analyze the level of finish line distinctness (FLD), and finish line accuracy (FLA), in 7 intraoral scanners (IOS) and one conventional impression (IMPR). Furthermore, to assess parameters of resolution, tessellation, topography, and color.

**Methods:**

A dental model with a crown preparation including supra and subgingival finish line was reference-scanned with an industrial scanner (ATOS), and scanned with seven IOS: 3M, CS3500 and CS3600, DWIO, Omnicam, Planscan and Trios. An IMPR was taken and poured, and the model was scanned with a laboratory scanner. The ATOS scan was cropped at finish line and best-fit aligned for 3D Compare Analysis (Geomagic). Accuracy was visualized, and descriptive analysis was performed.

**Results:**

All IOS, except Planscan, had comparable overall accuracy, however, FLD and FLA varied substantially. Trios presented the highest FLD, and with CS3600, the highest FLA. 3M, and DWIO had low overall FLD and low FLA in subgingival areas, whilst Planscan had overall low FLD and FLA, as well as lower general accuracy. IMPR presented high FLD, except in subgingival areas, and high FLA.

Trios had the highest resolution by factor 1.6 to 3.1 among IOS, followed by IMPR, DWIO, Omnicam, CS3500, 3M, CS3600 and Planscan. Tessellation was found to be non-uniform except in 3M and DWIO. Topographic variation was found for 3M and Trios, with deviations below +/− 25 μm for Trios. Inclusion of color enhanced the identification of the finish line in Trios, Omnicam and CS3600, but not in Planscan.

**Conclusions:**

There were sizeable variations between IOS with both higher and lower FLD and FLA than IMPR. High FLD was more related to high localized finish line resolution and non-uniform tessellation, than to high overall resolution. Topography variations were low. Color improved finish line identification in some IOS.

It is imperative that clinicians critically evaluate the digital impression, being aware of varying technical limitations among IOS, in particular when challenging subgingival conditions apply.

## Background

Intraoral scanners (IOS) have been available for over thirty years with a rapid increase in the number of commercially available systems in the last decade [1–6]. With what appears to be a shift in technology, several IOS have moved from monochromatic image acquisition, with or without coating, to systems with color video acquisition [4, 5, 7].

3D Compare Analysis, a method superimposing two surfaces after best-fit-alignment, has been adopted from engineering and used in several in vitro, and limited in vivo studies to evaluate IOS and conventional impressions (IMPR) [6–20]. Some studies have used terminology based on ISO standard, ISO 5727 [21]. However, we use a definition applied in engineering and metrology, defining accuracy as ‘the ability of a measurement to match the actual value’, a term used similarly to the ISO 5725 ‘trueness’. Precision was defined as ‘the ability of a measurement to be consistently reproduced’ and carries comparable meaning to precision used by ISO 5725.

To evaluate accuracy, surfaces of a physical model are commonly scanned with a reference scanner to which digital and analogue scans can be compared [6–14]. However, most of those studies compare the full surface of a preparation or teeth, and do not evaluate specific areas.

A different assessment of IOS and IMPR is through analysis of the marginal fit in final restorations. Although not necessarily having an additive effect, such analysis quantifies and sums all errors deriving from the digital or conventional impression, to manufacturer process and the eventual seating of the restoration. Several studies assessing the marginal fit of ceramic crowns has been accounted for in a review, displaying a non-significant misfit of 63.3 μm, (95% CI: 50.5–76.0 μm), for restorations from IOS and 58.9 μm, (95% CI: 41.1–76.7 μm), for restorations manufactured from IMPR [22]. Another review has shown similar results for single unit and short fixed dental prosthesis [23]. These findings serve as a numerical value to which IOS and IMPR, as an integrated part of the workflow, can be compared. Furthermore, the results are well within the commonly accepted 120 μm for good clinical fit postulated nearly five decades ago [24].

However, for IOS to reach wide clinical acceptance, it is essential that IOS perform equally well or better than scans of gypsum models deriving from conventional impressions, especially when the treatment consists of a fixed prosthesis [18, 23]. An area where clinical difficulty has been reported, is the scanning of subgingival areas and regions with localized bleeding [18].

IOS use varying acquisition techniques and software algorithms with system-specific characteristics of the resulting mesh [9]. Apart from accuracy, variations in a triangle mesh can be analyzed through resolution (triangle density), tessellation (level of triangle regularity) and topography (variations in height) [9, 25]. These system-specific variations may further affect the possibility of identifying the finish line and proper placement of the planned margin of the final restoration, a critical step in crown and bridge manufacturing. This is especially the case in subgingival situations where limitations of the specific scanner technology, combined with limited access and scanning angle, may result in a higher level of interpolation of scan data [9, 18]. This may have been overlooked in previous studies when evaluating scanners based solely on parameters of general accuracy, as specific localized deviations only make a small part of the overall dataset.

The aim of this study was to visualize any differences in finish line distinctness and finish line accuracy of IOS and IMPR in a preparation with a supragingival and subgingival finish line. Furthermore, to analyze specific IOS regarding mesh resolution, tessellation, topography, and the effect of color. Finish line distinctness was defined as the degree of visual clarity and identifiability in the reproduction of the finish line compared to a reference scan. Finish line accuracy was defined as the ability of a measurement to match the actual value of a reference scan in the immediate proximity to the finish line.

The null hypothesis of this descriptive study was that no sizeable differences exist between IOS systems and IMPR regarding finish line distinctness and finish line accuracy, indifferent of mesh properties.

## Methods

### ATOS reference-scan

A non-unibody model with screw-attached teeth to a hard gingiva model, (Model M-860MQD; Colombia Dentoform Corp, New York, USA), was used to mimic difference in color of tooth and gingiva and the physical separation of tooth and supporting structures seen clinically. This is especially the case in the area close to the finish line, where subsurface scattering may lead to light travelling through different media and thus resulting in light being emitted at different points and angles [26].

**Fig. 1:**
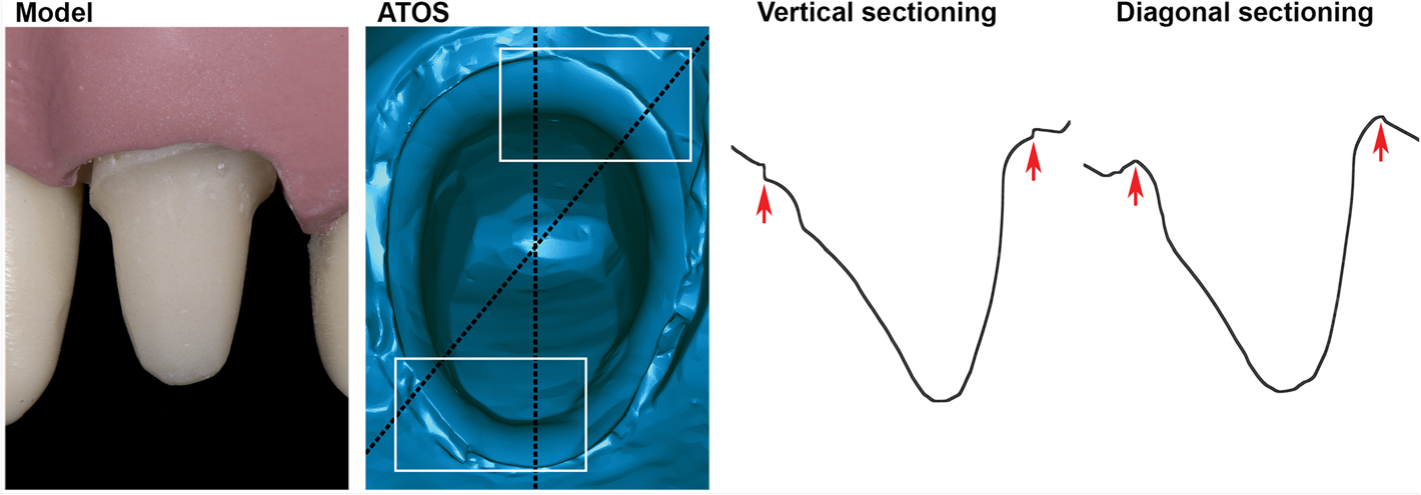
Buccal view of model with partially supra- and subgingival preparation and OVA of ATOS reference-scan. Rectangular demarcations depicting enlarged areas with subgingival finish line: DB (distobuccal), upper, MP (mesiopalatal), lower. Dotted lines show vertical and diagonal sectioning with respective 2D view

A preparation for a cemented crown was performed on a left upper lateral. The finish line was generally supragingival with two specific subgingival areas, distobuccal (DB), and mesiopalatal (MP), where the finish line was placed at the bottom of the sulcus (Fig. 1). Due to the partially translucent properties of the model, a thin layer of titanium dioxide coating, (Kronos Titandioxide; Kronos International Inc., Leverkausen, Germany), was applied with an airbrush (Iwata HP-TR1; Iwata Medea, Inc., Portland, USA). The model was scanned, (Cascade Control AB; Mölndal, Sweden), with an industrial-grade scanner (ATOS), (ATOS Triple Scan III 8MP resolution; GOM, Braunschweig, Germany), calibrated and tested according to VDI protocol (VDI e.V.; Düsseldorf, Germany). The scanner was operated by proprietary software, (ATOS Professional version 8.1; GOM) and reference-scan exported in STL file format after polygonization with details set to high.

### IOS and IMPR

Six manufacturers agreed to provide in total seven scanners for testing: 3M True Definition (3M), Carestream CS3500 (CS3500), Carestream CS3600 (CS3600), Dentalwings Intraoral Scanner (DWIO), Omnicam (OMNI), Planscan (PLAN) and Trios (TRIOS). Table 1 lists system, manufacturer, software version, light source, color/monochromatic acquisition and scanning technology.

**Table 1:** Intraoral systems, manufacturer, software versions and type of technology

System	Manufacturer	Software	Light Source	Color	Acquisition
3M True Definition	3M	2.0.2.0	LED	monochrome	Video
CS3500	Carestream	1.2.6.40	LED	non-true color	Image
CS3600	Carestream	2.1.6.30	LED	non-true color	Video
DWIO	Dental Wings	3.7.0.26	LED	monochrome	Video
Omnicam (CEREC)	Sirona	4.3	LED	non-true color	Video
Planscan	Planmeca	5.6.0.51	Laser (LED)	non-true color	Video
Trios 3	3Shape	1.3.4.5	LED	true color	Video

The reference-model was scanned with ten repetitions for each of the participating IOS systems per manufacturer protocol by an experienced clinician. The model was gently cleaned, (Quick Stick microbrushes; Dentonova AB, Stockholm, Sweden), from coating for reference-scanner ATOS, 3M and DWIO. Due to the model requirements of separate entities of teeth and the supporting structures, only one impression, (Impregum Penta H DuoSoft and Impregum Garant L Duosoft; 3M), with a tray (Position Tray; 3M), was taken as there was a risk of a position-shift of the screw-retained prepared tooth upon removal of the impression. The impression was treated with disinfectant, (MD 520; Dürr Dental AG, Bietigheim-Bissingen, Germany) for five minutes, air dried, and poured after 24 h with type IV dental stone (Fujirock EP; GC Europe, Leuven, Belgium). The gypsum model was scanned without sectioning using 3Shape D1000 (3Shape Dental System, Copenhagen, Denmark) with proprietary software, (version 16.4.0) to generate a 3D model (IMPR). All scans, impression and manufacturing of gypsum model were performed at room temperature (+ 20 to + 22 °C).

STL files were exported from proprietary scanning software for CS3500, CS3600, DWIO and PLAN, from 3M Online Case Manager for 3M, and with specific proprietary dental laboratory software for OMNI, (InLab 15; Sirona, Salzburg, Austria), TRIOS and dental laboratory scanner 3Shape D1000, (Dental System; 3Shape). After assessing the stabilization of the acquisition method, the tenth file of each IOS system was selected for further analysis after inspecting and verifying overall surface consistency, finish line, resolution, tessellation and topography with other scans within the same IOS group.

### Imaging and 3D compare analysis

The ATOS reference, the tenth IOS file of each system, and the IMPR file were imported into 3D inspection and metrology software, (Geomagic Control 2015; 3D Systems, Rock Hill, USA). High resolution snapshots were exported of the surface rendering. All snapshots were captured from a consistent predefined occlusal viewing angle (OVA). Figure 1 shows the ATOS reference-scan in OVA. Rectangular areas display subgingival distobuccal (DB) and mesiopalatal (MP) areas. Two sections, vertical and diagonal, visualize the preparation in 2D view.

The ATOS reference-scan was manually cropped along the finish line (ATOS PREP) in Geomagic Control for further 3D Compare Analysis. Although specific tools for detecting finish lines were available in some IOS workflows, several systems relied on third-party dental CAD software for that purpose. To obtain an identical workflow and conditions, such as artificial lighting, surface rendering algorithms and OVA, the finish line cropping of IOS and IMPR were performed in Geomagic after a best fit alignment versus the ATOS PREP. Display properties of ATOS PREP were set to only outline the finish line, allowing for manually tracing each superimposed IOS and IMPR file in OVA. Due to triangle size variations along ATOS PREP, inclusion or exclusion of IOS triangles crossing the ATOS PREP finish line were individually selected at great magnification. The exclusion criteria were that if more than half the estimated triangle area as seen from OVA was outside the ATOS PREP finish line, the triangle would be excluded and cropped from the IOS preparation.

3D Compare Analysis was performed on IOS and IMPR. High resolution snapshots were exported with deviation histograms at OVA, with setting at nominal ±25 μm and critical ±100 μm. Enlarged snapshots of 3D Compare Analysis in DB and MP areas with deviation histograms were also taken in OVA. A secondary deviation histogram was enabled with a “go/no go” setting of ±50 μm and snapshots were exported for scanners displaying deviations above that threshold: 3M, CS3500, DWIO, OMNI and PLAN.

### Image processing and analysis

Software snapshots were imported in Adobe Photoshop (version 2017.1.1; Adobe Systems Inc., San Jose, USA) using layers. The areas in IOS displaying deviations above ±50 μm were selected and the edges of the area demarcated with a filter creating an outline (stroke = 10 px). The demarcation outline was superimposed over the equivalent 3D Compare Analysis snapshot with deviation histogram to visualize the extent of the deviations from the finish line. The combined image displayed both the nominal ±25 μm and critical ±100 μm deviation mapping, as well as demarcated areas where deviations exceeded ±50 μm. Manual measurements were performed in Geomagic Control from the finish line to the demarcation with an estimated perpendicular angle to axial wall of the preparation as seen in OVA. As this measurement was manually selected, a certain amount of measurement error was expected, however, the measurement serves as an indication of the extension of the misfit from the periphery of the preparation above ±50 μm.

To evaluate the effect of color, proprietary software was used to create screenshots as not all IOS supported color export. For CS3600 and PLAN a proprietary viewer was used, and for OMNI and TRIOS screenshots were taken in proprietary dental laboratory software. The angle was manually set to display an occlusal view, however, an exact congruence in angulation similar to OVA, artificial lighting and color settings, could not be fully achieved.

## Results

Several of the systems display similarities which makes it challenging to individually rank IOS and IMPR. However, there are variations attributed to specific scanners where there is room for a clear separation based on image analysis.

**Fig. 2:**
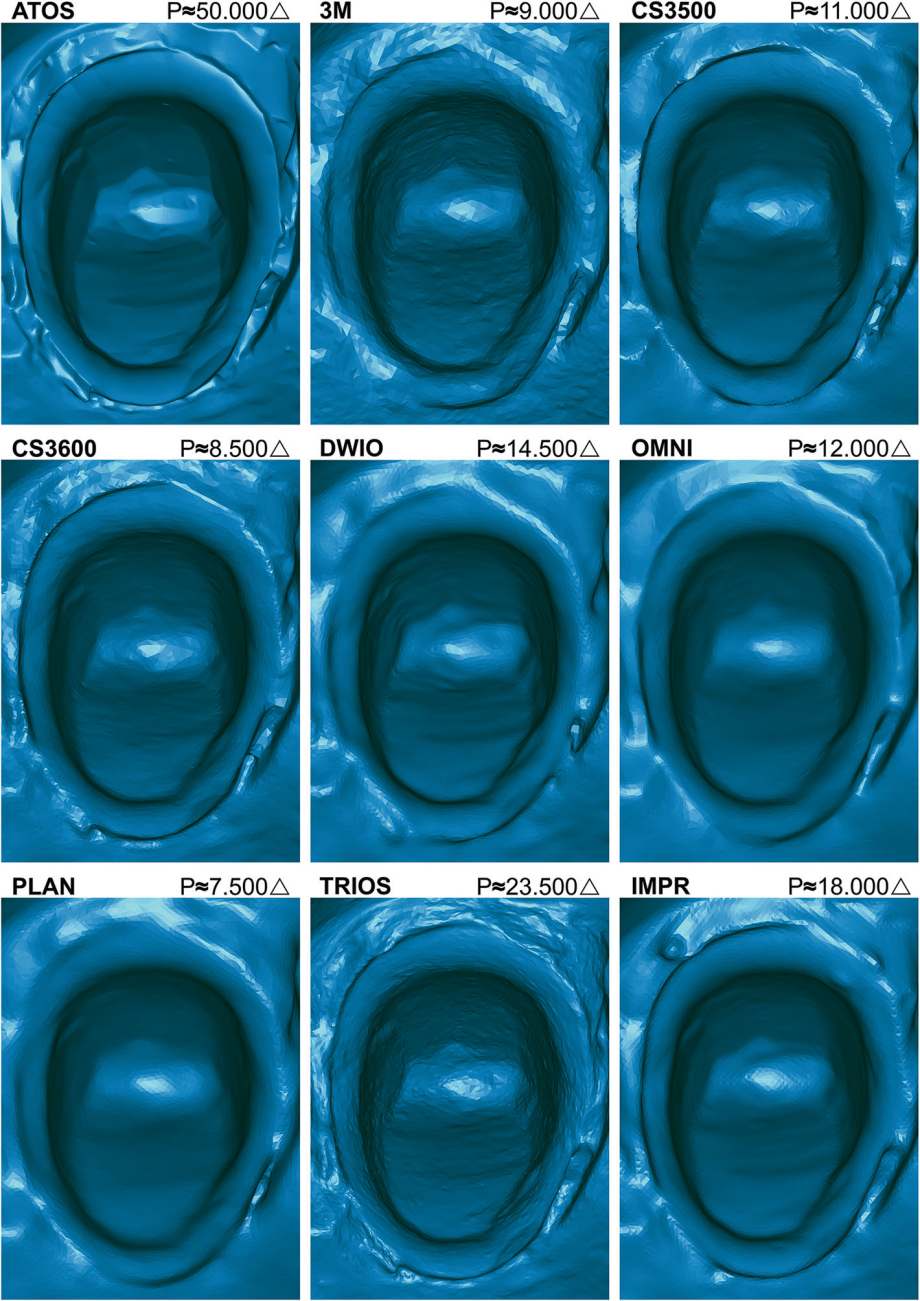
Comparison of rendered surface and circumferential finish line distinctness in OVA for ATOS, IOS and IMPR. Triangle count (P) refers to the full preparation without surrounding soft tissues

Figure 2 shows a rendered view for ATOS and each IOS and IMPR and the triangle count for the cropped preparation. The ATOS reference-scanner presented a resolution of 50.000 triangles, followed by rounding to nearest five-hundred: TRIOS (23.5000), IMPR (18.000), DWIO (14.500), OMNI (12.000), CS3500 (11.000), 3M (9000), CS3600 (8.500) and PLAN (7.500). TRIOS had a triangle count of 1.6–3.1 times higher than other IOS and 1.3 times higher than the laboratory scanner for IMPR. When comparing the circumferential finish line distinctness with ATOS reference-scan, TRIOS shows the highest overall distinctness. Systems displaying finish line distinctness at the lower end were PLAN, DWIO and 3M, where the latter were less distinct in subgingival DB and MP areas predominantly. Both PLAN and 3M showed the lowest triangle count, whereas DWIO had the second highest count among IOS.

**Fig. 3:**
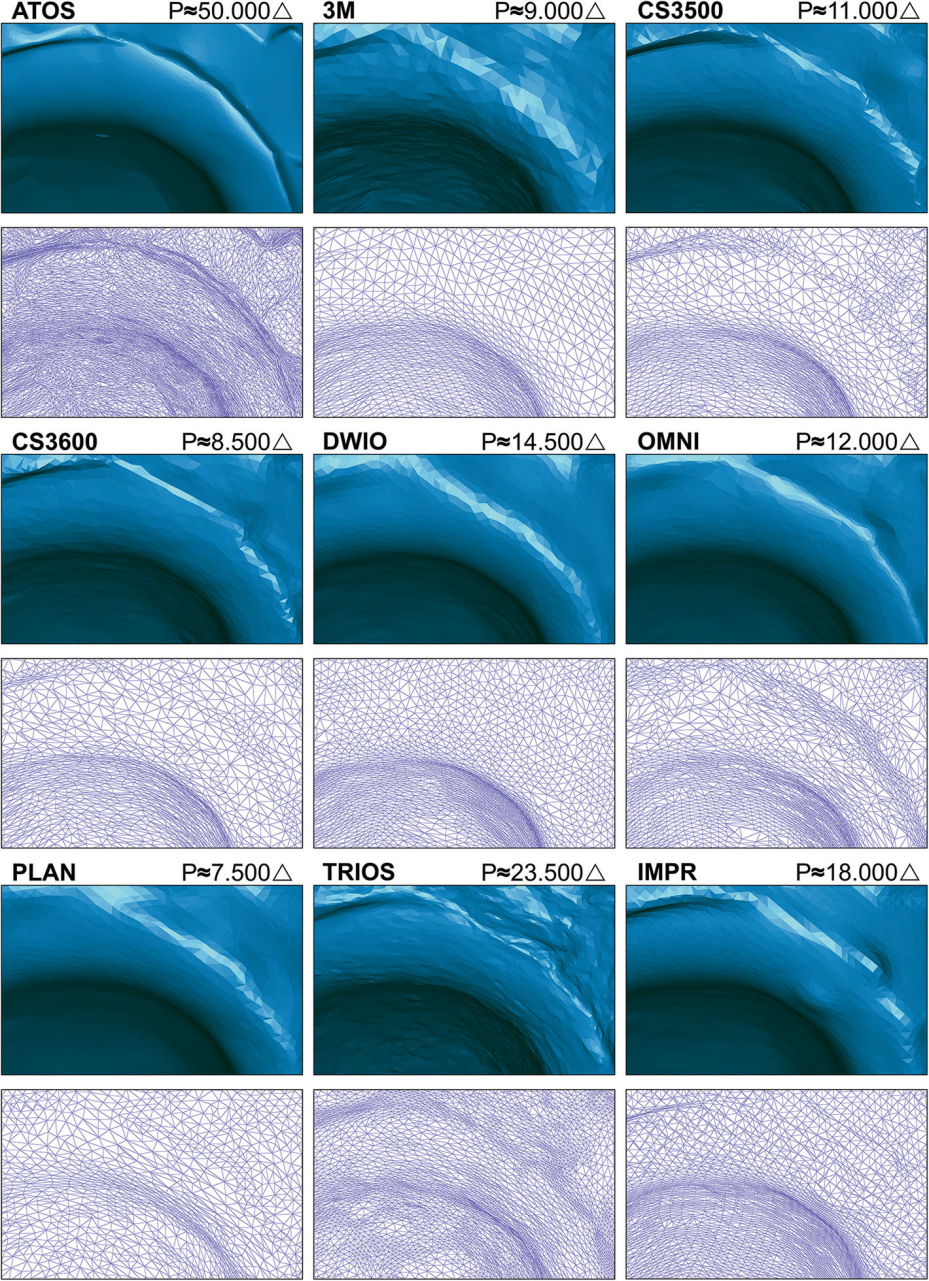
Comparison of rendered surface and 3D mesh displaying finish line distinctness in OVA for DB (distobuccal) area in ATOS, IOS and IMPR. Mesh displays varying tessellation and inter-system relative triangle size. Triangle count (P) refers to the full preparation without surrounding soft tissues

Figure 3 displays a comparison of enlarged area DB in OVA with surface rendering and underlying 3D mesh. The system showing the highest finish line distinctness was TRIOS, both in rendering and mesh view. CS3600 appeared to have a similar finish line distinctness to its predecessor, CS3500, however, the finish line area suffered somewhat from the low resolution and presented larger faceting than TRIOS. OMNI had a consistent finish line distinctness, but lacked some of the distinctness seen in TRIOS, as it carried a rounded finish line transition. The scanner with the lowest finish line distinctness was 3M, where both rendering, and mesh were lacking distinctness.

The mesh of 3M and DWIO showed a higher level of uniformity in tessellation close to the location of the finish line, as opposed to other systems which through their variations in triangle size increased the resolution in areas with undulations to better depict transitions.

**Fig. 4:**
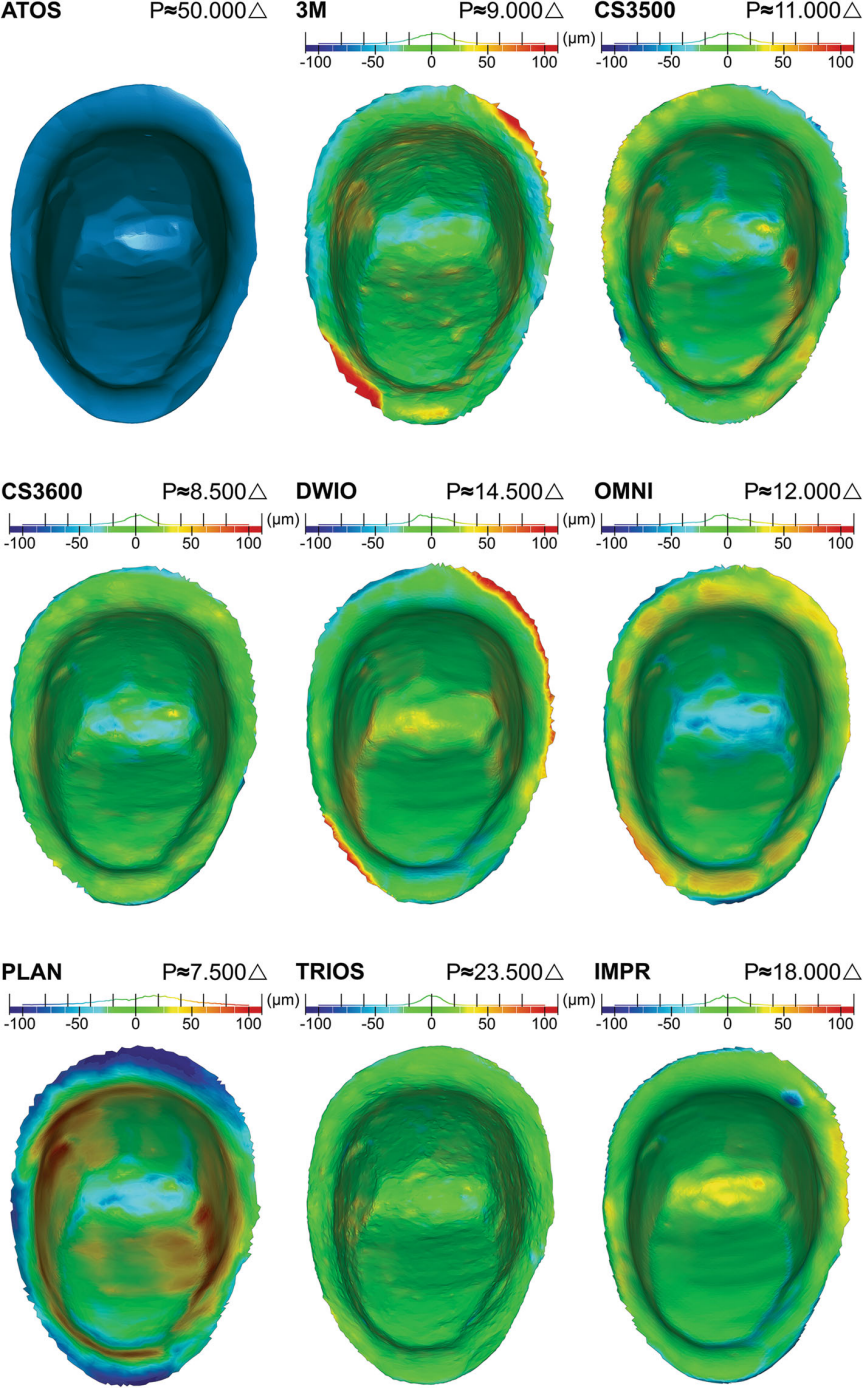
Comparison of full preparation in OVA of rendered ATOS PREP and 3D Compare Analysis for IOS and IMPR. Histogram settings nominal ±25 μm and critical ±100 μm displaying general accuracy, finish line accuracy and topographic noise. Triangle count (P) refers to the full preparation

Figure 4 displays ATOS PREP rendering and the 3D Compare Analysis for IOS and IMPR. The deviation can be analyzed in the histogram which shows an even distribution of most deviations within the nominal area ± 25 μm. However, the scanner which did not conform was PLAN, displaying overall deviations, but particularly in the finish line area. Furthermore, 3M, DWIO and to some extent OMNI, displayed deviations in subgingival DB and MP areas. The systems showing the highest overall accuracy based on color deviation evaluation and distribution of deviations in histogram, were primarily TRIOS and CS3600.

Image analysis revealed some topographic noise in 3M and TRIOS that were not visible in other systems. 3M displayed somewhat larger specks that reached above ±25 μm, whilst the TRIOS system appeared as minor noise limited to below ±25 μm.

**Fig. 5:**
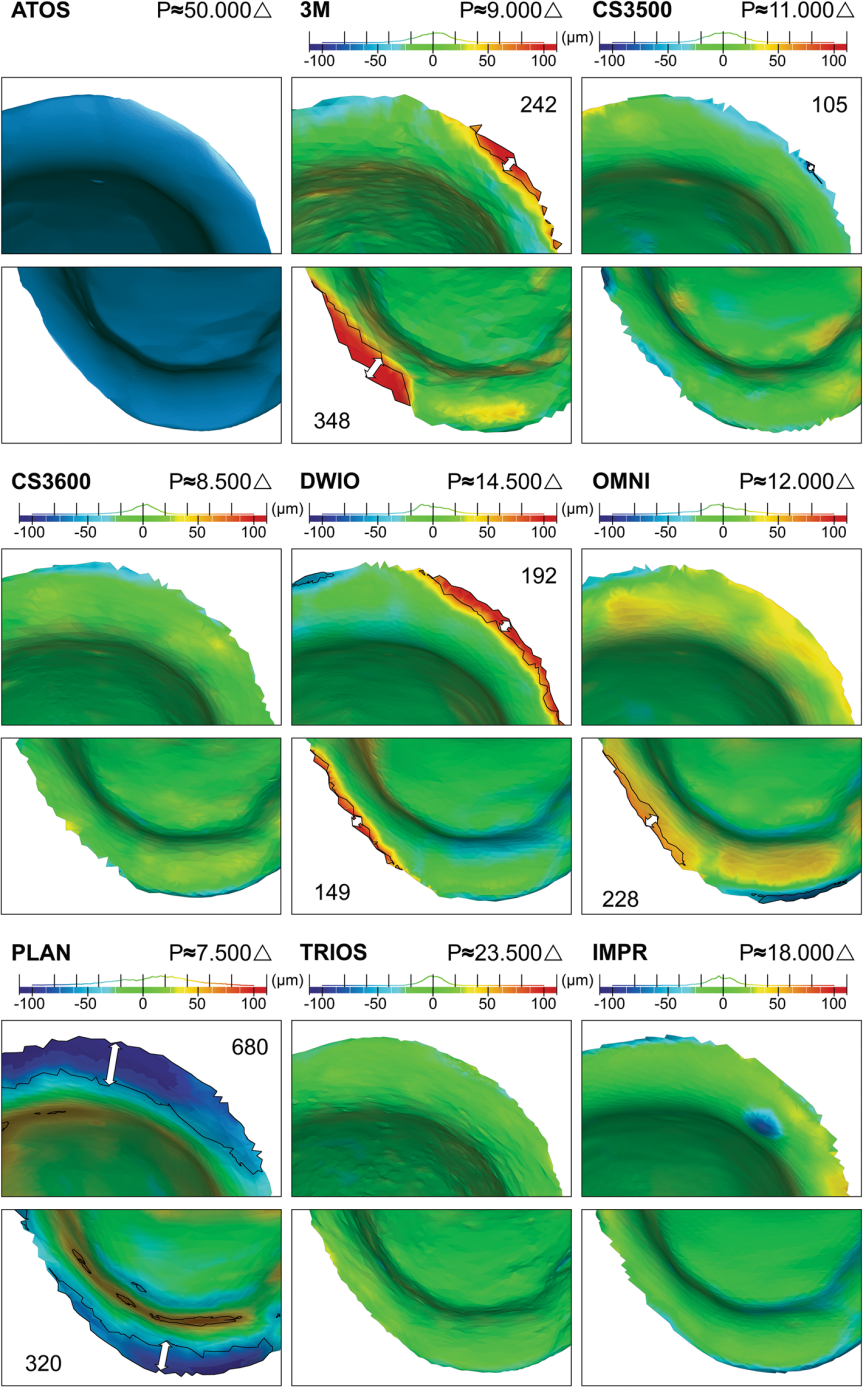
Enlarged OVA of rendered ATOS PREP and 3D Compare Analysis in DB (distobuccal) and MP (mesiopalatal) area. Histogram settings nominal ±25 μm and critical ±100 μm displaying finish line accuracy. Demarcation depicts areas with deviations above ±50 μm. Arrows with respective measurements display distance from finish line to demarcation line. Triangle count (P) refers to the full preparation

Figure 5 shows the enlarged DB and MP area of the ATOS PREP rendering and 3D Compare Analysis of IOS and IMPR. Measurements indicate the longest distance from the finish line towards the axial wall of the preparation where deviations reach above ±50 μm. The system with the highest finish line accuracy were TRIOS and CS3600, both systems showed deviations below ±25 μm. IMPR displayed deviations above ±50 μm at the periphery, but failing to reach more than 30–50 μm from the finish line. CS3500 showed a small and localized negative deviation measuring 105 μm from the periphery, DWIO a positive deviation of 192 μm from the periphery, 3M a positive deviation of 348 μm from the periphery, whilst PLAN displayed a larger negative deviation of 680 μm from the periphery. Measurement of OMNI reached 228 μm from the periphery, however, the deviation was in the lower range within + 50 to + 70 μm as opposed to DWIO, 3M and PLAN reaching well above the critical histogram level of ±100 μm.

Comparing the edge of the cropped preparation revealed that the smoothness of the finish line varied between systems where TRIOS showed the highest level of smoothness, and systems with lower resolution had a higher level of jaggedness.

**Fig. 6:**
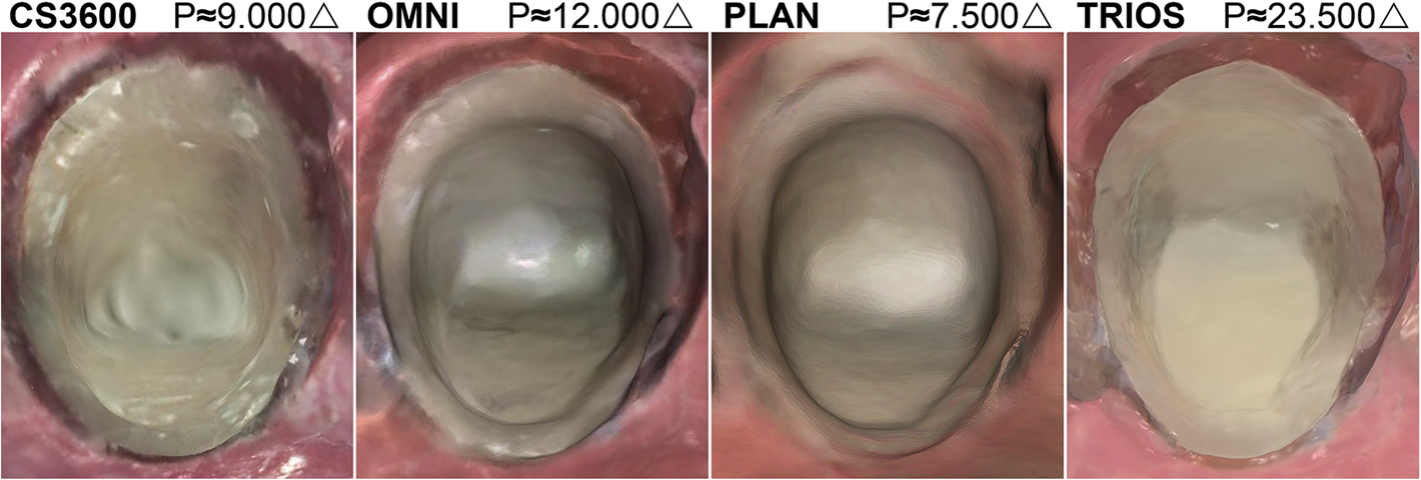
Variations of color rendition quality in proprietary software in occlusal view. Triangle count (P) refers to the full preparation

Figure 6 displays a comparison of color screenshots in CS3600, OMNI, PLAN and TRIOS. Apart from PLAN, with a low finish line distinctness and color bleed, the color rendition offered a contrast that may assist in identifying the finish line compared to the monochromatic STL files shown in Fig. 2.

## Discussion

Previous in vitro studies evaluating IOS and IMPR vary considerably for study design, but also regarding material properties of the reference-model [15, 18]. Nevertheless, extensive literature reviews show results which are clinically satisfactory for both digital and analogue impressions, as did the full manufacturing flow of single restorations and shorter fixed prosthesis [18, 22, 23].

However, we have noticed when working clinically with multiple IOS in parallel, that there are large variations in distinctness of the finish line of the acquired scans, and that IOS and desktop scanners display unique system-specific mesh appearances. The aim of the present in vitro study was to evaluate any differences of finish line distinctness and finish line accuracy of IOS and IMPR, a critical component in prosthodontics which has not been investigated previously. Furthermore, the descriptive method aimed at visualizing the effect of other parameters, such as mesh resolution, tessellation, topography, and the effect of color.

The results of this study do not support the null hypothesis that there were no sizeable differences between IOS and IMPR regarding finish line distinctness and finish line accuracy.

This in vitro-study adopts a test model where the digital and conventional impressions were taken under the best conditions without interference from extrinsic adverse factors, such as gingival crevicular fluid, blood, or displaced retraction cords. The preparation, with supragingival finish line and two areas simulating subgingival finish lines, was selected to investigate the IOS limitations as it imposes a great challenge for successful identification of the finish line [18].

TRIOS, with the highest triangle count, displayed the highest level of finish line distinctness and together with CS3600, the highest finish line accuracy and surpassed IMPR. DWIO and PLAN on the other hand displayed a generally low level of finish line distinctness and finish line accuracy. Together with 3M, deviations in local subgingival areas reached deviations above ±100 μm. This deviation in IOS relates by at least a two-fold factor to that seen in studies on margin fit of final restorations, which also take in consideration all contributing factors, such as the milling of the restoration and the seating [22]. Hence, the deviations should be considered sizeable in relation to the full workflow.

PLAN showed the lowest finish line accuracy of all IOS, as well as the lowest overall accuracy, and contrary to other IOS, held negative deviations. A positive deviation may result in a restoration being short onto the preparation and with a potential larger spacing. A large negative deviation may result in a restoration having premature contact in specific hotspots, thus leaving greater spacing in other areas. It appears that the acquisition method by laser and triangulation technology used in PLAN suffers the same deficiencies previously found in the preceding E4D system [9].

Deviations above ±50 μm reached varying distances from the periphery in 3M, DWIO and predominantly PLAN. The size of deviations in combination with the extent of the deviations from the finish line may play an important part in the long-term success of final restorations.

Resolution varied between the evaluated systems, with PLAN showing the lowest triangle count, as well as a low level of finish line distinctness and the lowest finish line accuracy. DWIO on the other hand, with the second highest resolution, also showed a low level of finish line distinctness and low finish line accuracy. The highest triangle count was found in the TRIOS system, which also had the highest level of finish line distinctness. However, the scanner with the second lowest triangle count, CS3600, showed a similar degree of local finish line accuracy as TRIOS. Thus, overall resolution appears to not have a direct relation to the finish line distinctness and finish line accuracy, but may depend on localized finish line resolution. These findings are in agreement with previous studies [9, 25].

The effect of low resolution was however visible in the cropped area of the finish line with a higher level of jaggedness. It is unclear how proprietary software as well as different dental CAD software treats the jaggedness through post-processing and possible triangle subdivision when plotting the finish line. The system with the highest level of smoothness was TRIOS.

Tessellation of any 3D mesh derives from both the specific scanning technology and from active engineering choices when designing software algorithms. Although 3M and the DWIO had different mesh appearances, a consistent higher uniformity was present in the tessellation at the finish line when compared to other IOS and IMPR, (Fig. 3). This may not have an impact on larger surface accuracy, but can be perceived when evaluating the finish line distinctness which holds a low resolution and lacks the capacity to clearly depict the undulating transition area. Poor depiction of the finish line may lead to an unnecessary over- or undersized contour of the final restoration.

Topography describes the variations in height of a surface. A previous study has shown that earlier systems based on coating displayed a smoother topography, whilst non-coating systems with a higher resolution produced a surface with greater noise [9]. Although many of the scanners in this study belong to a newer generation, the non-coating TRIOS system displays some minor noise not seen among the other non-coating IOS and is dependent on the specific technology. However, the deviation spectrum in TRIOS was within the nominal range and most likely lacks any clinical effect in later processing and manufacturing. 3M displayed minor areas outside the nominal threshold.

The introduction of color among IOS systems, may improve the detection of the finish line due to the visible contrast between tooth and soft tissue as seen in Fig. 6 when compared to the monochrome renderings in Fig. 2. TRIOS and OMNI, and to some extent CS3600 showed a clear and distinct color rendering. PLAN using laser for measurements and RGB LED for color mapping, had low congruence with color bleed from the tooth onto the adjacent surface, thus not increasing the identification of the finish line.

Several described factors may influence the finish line distinctness and identification of the finish line. However, a parameter not simulated is the possibility to rotate the model in the 3D space and through variations of inter-facet angulations visualize variations in the 3D rendering. This rendition created with artificial lighting, generates glare, light reflection of high to low intensity as well as full cut-off, and can assist the operator in identifying a distinct finish line. Furthermore, tools in proprietary software and third party dental CAD/CAM solutions can facilitate and automate the recognition of the finish line, and use 3D imaging snapshots to enhance the manual identification [7]. However, these tools only enhance existing features of the 3D mesh and does not replace a high-quality scan.

There are several limitations within this study. First, the need for coating the translucent preparation model with titanium dioxide to allow for a reference-scan. Even though thickness of the coating material was minute, a material buildup could occur [18]. To limit this negative impact, the reference scanning was outsourced to a specialized entity, with extensive experience of scanning for the industry in general, and for research and development within leading dental companies. Specific airbrush technology with fine-adjustable air-pressure was used to deliver the thin coating at control beyond that of aerosols or powder dispenser used in the field of dentistry.

Second, as only one file (the tenth scan/repetition) for each IOS was investigated regarding finish line accuracy, the deviations may fluctuate both in severity and distance from the finish line. However, it is beyond the scope of this descriptive pilot study to assess the full intra-system range of such deviations or the precision of each system.

Last, the used in vitro model cannot fully simulate hard and soft-tissue interaction, and it excludes adverse factors known to negatively affect the quality of impressions. Thus, the clinical reality may prove to be more challenging than conditions in this study.

From a clinical perspective, it is essential that IOS can perform well in all scenarios, with similar, or better results than conventional impressions. This study shows that some investigated IOS can provide finish line distinctness and finish line accuracy that is higher than IMPR in vitro. However, not all IOS performed equally well.

## Conclusions

This study shows that there are sizeable variations between IOS with both higher and lower finish line distinctness and finish line accuracy than IMPR. High finish line distinctness was more related to high localized finish line resolution and non-uniform tessellation, than to high overall resolution. Topography variations were generally low. Color output from some scanners may enhance the identification of the finish line due to contrasting colors, but is dependent on the underlying technology.

It is imperative that clinicians critically evaluate the digital impression, being aware of technical limitations and system specific variations among IOS, in particular when challenging subgingival conditions apply.

## Abbreviations

3D: Three dimensional
CI: Confidence interval
FLA: Finish line accuracy
FLD: Finish line distinctness
IMPR: Impression
IOS: Intraoral scanners
